# The first research agenda for the chiropractic profession in Europe

**DOI:** 10.1186/2045-709X-22-9

**Published:** 2014-02-10

**Authors:** Sidney M Rubinstein, Jenni Bolton, Alexandra L Webb, Jan Hartvigsen

**Affiliations:** 1 Department of Health Sciences, Faculty of Earth and Life Sciences, VU University, Amsterdam, 1081 HV Amsterdam, The Netherlands; 2 Department of Research and Graduate Studies, Anglo-European College of Chiropractic, Bournemouth, UK; 3 Centre for Learning Anatomical Sciences, Faculty of Medicine, University of Southampton, Southampton, UK; 4 Institute of Sports Science and Clinical Biomechanics, University of Southern Denmark, Odense, Denmark and Nordic Institute of Chiropractic and Clinical Biomechanics, Odense, Denmark

**Keywords:** Chiropractic, Research, Consensus, Policy making, Delphi technique

## Abstract

**Background:**

Research involving chiropractors is evolving and expanding in Europe while resources are limited. Therefore, we considered it timely to initiate a research agenda for the chiropractic profession in Europe. The aim was to identify and suggest priorities for future research in order to best channel the available resources and facilitate advancement of the profession.

**Methods:**

In total, 60 academics and clinicians working in a chiropractic setting, and who had attended any of the annual European Chiropractors’ Union/European Academy of Chiropractic (ECU/EAC) Researchers’ Day meetings since their inception in 2008, were invited to participate. Data collection consisted of the following phases: *phase 1* identification of themes; *phase 2* consensus, which employed a Delphi process and allowed us to distill the list of research priorities; and *phase 3* presentation of the results during both the Researchers’ Day and a plenary session of the annual ECU Convention in May 2013. In addition, results were distributed to all ECU member countries.

**Results:**

The response rate was 42% from phase 1 and 68% from phase 2. In general, participants were middle-aged, male and had been awarded a Doctor of Philosophy (PhD) as well as chiropractic degree. Approximately equal numbers of participants had obtained their chiropractic degree from the UK/Europe and North America. The majority of participants worked primarily in an academic/research environment and approximately half worked in an independent institution. In total, 58% of the participants were from the UK and Denmark, collectively representing 44% of the chiropractors working in Europe. In total, 70 research priorities were identified, of which 19 reached consensus as priorities for future research. The following three items were thought to be most important: 1) cost-effectiveness/economic evaluations, 2) identification of subgroups likely to respond to treatment, and 3) initiation and promotion of collaborative research activities.

**Conclusions:**

This is the first formal and systematic attempt to develop a research agenda for the chiropractic profession in Europe. Future discussion and study is necessary to determine whether the themes identified in this survey should be broadly implemented.

## Background

Research involving chiropractors is evolving and expanding in several European countries while resources are limited. At the European Chiropractors’ Union/European Academy of Chiropractic (ECU/EAC) Researchers’ Day in Zurich, Switzerland (June 2011), it was decided amongst the members that we needed to establish a vision for chiropractic research in Europe for the forthcoming five to ten years. This is in line with the results from a recent survey of all ECU member nations by the EAC Research Council [1]. Various other initiatives have also been conducted within the chiropractic profession, which include a strategic planning conference whose goal was to better service the public and at the same time promote the profession [2].

There are three primary reasons why the instigation of a research agenda is believed to be important. Firstly, it is thought that the process could facilitate unity within the European chiropractic research community and encourage collaboration on research items considered to be important. Secondly, no European chiropractic research agenda has ever been established, which is in contrast to North America where an agenda was first established in 1997 [3] with an update in 2006 [4,5]. Finally, researchers with chiropractic backgrounds frequently work and publish with other professionals, so it is of interest to investigate whether priorities from a chiropractic research agenda differ from other published agendas. For example, recently, a set of research priorities was established in the UK for non-pharmacological therapies for common musculoskeletal problems through a consensus process [6].

The goal of this study was to establish a list of suggested research priorities for the European chiropractic profession. In order to investigate this, a Delphi approach was used. The Delphi procedure is a methodology designed to obtain consensus from a panel of experts on issues or questions that are “shrouded in uncertainty, but cannot be measured or evaluated in the classical sense” [7]. This is typically achieved through a series of rounds where information is fed back to panel members using questionnaires and has been used extensively in social science research [8].

## Methods

### Selection of panel members

Academics and clinicians, who had attended any of the ECU/EAC Researchers’ Day meetings since their inception in 2008, were invited to participate. The Researchers’ Day is an annual meeting designed to bring researchers from chiropractic institutions and clinicians, involved with or interested in research, together in an informal setting and is always held on the day prior to the annual ECU Convention. The purpose of the Researchers’ Day is to share information and exchange ideas and provide those in attendance with a better idea of the current research being conducted by colleagues in the field. The meeting is a mix of presentations and workshops and has included 60 different participants over the past five years. The complete list of invited participants is available upon request from the primary author (SMR).

### Data collection

#### ***Phase 1: Identification of themes***

Data collection consisted of three phases. In the initial phase, participants were invited to participate. In the introductory letter, the purpose of the study was described, in addition to the steps involved in the process (Additional file 1: Appendix 1). Using an electronic survey (SurveyXact, developed by Rambøll Management Consulting company), participants were asked to list research topics they considered important for the chiropractic profession in Europe. The data collection tool was open-ended and participants were required to organize their suggestions around the following four domains: 1) epidemiological research, 2) clinical research, 3) basic science research, and 4) other. Participants were otherwise free to include whatever items they deemed relevant. The results were subsequently coded independently by two of the team members (SMR, JH) and these items were then discussed with all four members of the team. Decisions were then made which items to include based upon emerging themes related to the domains listed above. The methodological rigor and decisions made in this step can be viewed as analogous to qualitative research in which general themes are identified following open-ended questioning and items are ‘coded’ by the researchers [9]. From these results a list of items was created which the participants could rate in the second phase of the process.

#### ***Phase 2: Delphi process***

In the second phase, the structured list of domains with specific items identified in phase 1 were circulated online via SurveyXact to all 60 invited participants (whether they responded to the initial phase or not). In general, the questions were worded as follows: “Should more research be conducted on….”; “Should we examine…”; or “Should we investigate…” and subsequently, specific items or populations were listed. The questionnaires from all rounds are available in Additional file 1: Appendix 2 and Additional file 1: Appendix 3. Participants were asked to rate each item according to its importance on a 9-point ordinal scale ranging from 1 (‘extremely unimportant’) to 9 (‘extremely important’). This method, as well as the level of agreement regarding consensus (described below), is consistent with a recent Delphi study for the assessment of patients with low back-associated leg pain in primary care [10]. Participants were given three weeks to respond and were sent reminders where necessary.

### Consensus

The level of agreement between participants was set at 70%, which is consistent with previous study methods [10]. Items rated between 7 to 9 on the scale by 70% or more of the participants were classified as ‘important’, while items rated between 1 to 3 and 4 to 6 by 70% or more of the participants were classified as ‘unimportant’ and of ‘uncertain importance’, respectively. Disagreement for the same item was determined *a priori* when >30% rated an item ‘unimportant’ (1 to 3) and >30% rated an item ‘important’ (7 to 9). All other combinations in rating the items were considered to lack consensus.

During the process, items achieving consensus were made available to the participants so they could see which items had reached consensus. Participants were allowed to comment, but were excluded from further voting on these items. All items which did not achieve consensus remained in the document and were available for voting in the subsequent round. In addition, comments provided from the participants were discussed among the project team members and added to the subsequent rounds, when necessary (Additional file 1: Appendix 4). The decision to include a comment was based upon interpretation of the comment and whether it was thought to be a new and relevant item by the project team during the ensuing discussion, and was not based on the number of times the comment was suggested by the participants. An item was not included if there was repetition and/or the item had already reached consensus. The Delphi process continued until information saturation had been reached which was determined by the project team.

### Rating items

Following the final Delphi round, participants were asked to rate all 19 items reaching consensus using a 5-point scale, ranging from most (5 points) to least important (1 point). These items were tabulated and the items were ranked according to their score (highest to lowest) (Additional file 1: Appendix 5). In those cases where more than one item was rated equally, those items were assigned equal ranking. No subsequent attempts were made to assign a distinction between those particular items.

#### ***Phase 3: Presentation of the results at an annual European chiropractic forum***

Prior to the annual ECU Convention, the results were made available to the ECU Executive Council (which represents the governing Board of the ECU). The purpose for doing so was to inform the members of the results and give them the opportunity to reflect on these. At the convention, the results were presented at both the ECU/EAC Researchers’ Day in which the Executive Council and General Council (which includes representatives from each of the ECU member nations) were in attendance as well as during a plenary session at the convention in May 2013. The joint session which took place during the Researchers’ Day was specifically scheduled in order to allow members of the Executive Council (n = 5) and General Council (n = 16) who were present on that day to attend this meeting. During these meetings notes were taken by one of the project team members (AW) including all comments and questions raised.

### Data analysis

Sociodemographic data, which included age, gender, highest academic degree obtained, and chiropractic institution attended and current institutional affiliation (if relevant), were collected from the participants following the first round of phase 2. Dichotomous data are presented as a proportion and all continuous data are reported as mean (standard deviation). Frequencies of responses from the survey were examined in Excel (Microsoft Corporation, 2003). Results were compiled by one of the authors (SMR) and all responses were checked independently by a second author (AW) in order to ensure quality of the data.

### Ethics approval

Institutional Review Board approval was given by the VU University Medical Centre, Amsterdam, The Netherlands and is available upon request (project number 2012/083).

## Results

### Sociodemographic characteristics

The characteristics of the 46 participants (from the 60 invited) and who completed round 1 of phase 2, are listed in Table 1. In general, participants were middle-aged, male and had been awarded a Doctor of Philosophy (PhD) as well as a chiropractic degree. Approximately equal numbers of participants had obtained their chiropractic degree from the UK/Europe and North America. The majority worked primarily in an academic/research environment. In addition, 49% worked in an independent institution (e.g. Anglo-European Chiropractic College (AECC), Nordic Institute of Chiropractic and Clinical Biomechanics (NIKKB), Franco-European Institute of Chiropractic (IFEC)) whereas 34% worked in a university (e.g. University of Southern Denmark (SDU), University of Glamorgan, VU University Medical Centre, Amsterdam, while 17% did not have any academic affiliation. Furthermore, 41% of the participants were affiliated with one of two institutions (AECC or NIKKB).

**Table 1:** Sociodemographic characteristics of the participants (N = 60)

**Characteristic**	**Mean (SD)**	**Percentage (%)**
Age (yr.)	47 (7)	
Gender (% male)		64
Highest academic degree achieved		
PhD		49
MSc		29
Other (DC, BSc, MD)		22
Degree in chiropractic (% yes)		91
Country where chiropractic degree was received		
UK/Europe		49
North America		44
Australia		7
Primary place of work		
Academic		63
Clinical practice		30
Combination clinical practice + academic		4
Administration		2
University/institutional affiliation		
Anglo-European Chiropractic College (AECC)		28
No academic affiliation		17
Nordic Institute of Chiropractic and Clinical Biomechanics (NIKKB)		13
University of Southern Denmark (SDU)		7
Welsh Institute of Chiropractic, University of Glamorgan		7
Franco-European Institute of Chiropractic (IFEC)		5
VU University Medical Centre, Amsterdam		5
Other^1^		18

^1^The following universities/institutions represent <5% of the total:Karolinska Institute (3%), Orthopaedic University Hospital Balgrist (University of Zurich) (3%), University of Oslo (3%), Swiss Chiropractic Institute (3%), University of Stavanger (2%), National University of Health Sciences (2%), University of Southampton (2%).

### Percentage of registered chiropractors and participants in this study stratified by country

Table 2 contains an overview of the percentage of registered chiropractors within the ECU including Denmark (which is not a current member of the ECU) and the percentage of participants in this survey stratified by participating country. In total, 58% of the participants were from the UK and Denmark, while collectively those countries represent 44% of the chiropractors working in Europe. For the remaining countries represented in the survey, there did not appear any demonstrable differences between the percentage of working chiropractors in that country and representation in this survey. In total, 85% of the chiropractors registered with the ECU (including Denmark) were represented by at least one individual from that country. Countries that did not have representation included Cyprus, Finland, Germany, Hungary, Iceland, Ireland, Italy, Liechtenstein, Luxembourg, Poland, Spain and Turkey.

**Table 2:** **Percentage of chiropractors registered with the European Chiropractors’ Union (ECU) and percentage of participants in this survey by participating country**^**1**^

**Country**	**Chiropractors registered with the ECU (%)**^**2**^	**Participants in this survey (%)**
United Kingdom	31	38
Denmark	13	20
Norway	14	12
France	8	5
Switzerland	6	7
The Netherlands	6	7
Sweden	4	5
Belgium	2	2
Greece	1	2
USA	N/A	3
Countries not represented in this survey	15	N/A
**Total**	**100**	**101**^**3**^

^1^Denmark is not a member of the ECU, but has been added in the figures here. An estimated 550 chiropractors work in Denmark. (http://www.danskkiropraktorforening.dk/English/Chiropractic-in-Denmark/).

^2^Percentages have been rounded to whole numbers explaining why the total is 101%.

^3^Based upon known figures for the Spring 2013.

N/A = not applicable.

### Participation

**Figure 1:**
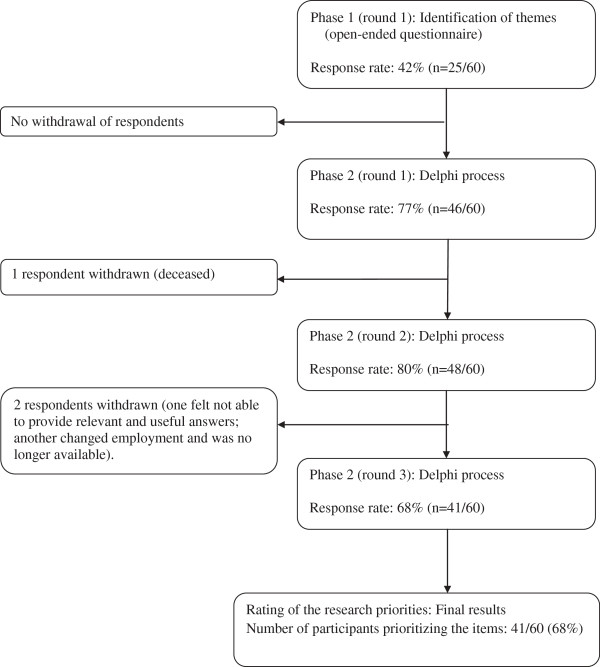
Flow chart of participation.

Participation during each phase of the study is depicted in Figure 1. Three rounds were necessary in phase 2 in order to reach consensus. This included the identification of consensus items, clarification where necessary and the addition of new items identified from participant comments in the previous round. The response rates were 42% (n = 25/60) and 68% (n = 41/60) for phases 1 and 2, respectively.

### Results from phase 1 & 2

In total, 44 items were identified from phase 1 and included in the survey (Table 3). In the first round of phase 2, 7 items reached consensus and were removed from further voting (Table 4). In addition, 26 items (Table 4) were added from the comments provided, and included for the subsequent round. Following this round, an additional 12 items reached consensus (see Table 4, column 3). Comments were provided in this round as well, but were not thought to influence the process or add anything new; that is, it was felt that information saturation had been reached. This marked the end of the study. In total, 70 items were identified during the process.

**Table 3:** Items identified during phase 1

**I. Epidemiological and clinical research**	
**A. Effects of treatment ** 1) Should more research be conducted in the following areas?	- Cost-effectiveness (i.e. conduct economic evaluations)
	- Maintenance care
	- Short-term effects (<3 months)
	- Sub-groups likely to respond to care
	- Safety/adverse events
	- Dose–response and frequency of treatment
	- Comparison of different chiropractic techniques
	- Other?
2) Should more research be conducted on chiropractic treatment effects and responses upon:	- Musculoskeletal pain?
	- Other?
3) Should more research be conducted on chiropractic treatment effects and responses in any of the following specific populations?	- Infants and babies
	- Pre-school and children
	- Adolescents
	- Geriatrics
	- Pregnant women
	- Athletes
	- Other
**B. Prognostic research ** 1) Should more prognostic research be conducted on the clinical course of musculoskeletal pain (for the identification of subgroups) in:	- A chiropractic population?
	- Population treated by other health care practitioners?
2) Should we examine any of the following specific factors as predictors of outcome with chiropractic treatment?	- Psychosocial factors
	- Clinical findings
	- Other
**C. Prevalence/incidence/prevention/population studies of musculoskeletal conditions** 1) Should we examine prevalence/incidence/prevention of musculoskeletal conditions in the following specific patient populations?	- Infants and babies
	- Pre-school and children
	- Adolescents
	- Geriatrics
	- Pregnant women
	- Athletes
	- Other
2) Should we examine the following specific topics?	- Determinants of work absenteeism for musculoskeletal conditions
	- Descriptive studies on clinics, chiropractors and patients in all ECU member countries
	- Risk factors for incidence of musculoskeletal pain
	- Prevention of musculoskeletal pain in primary and secondary care
	- Other
**D. Issues of chiropractic practice -** Should we examine:	- The clinician-patient relationship
	- Who are the care seekers and what triggers their care seeking
	- Other
**II. Basic science research**	
**A. Anatomy and physiology** 1) Should we investigate the anatomical and/or (neuro)physiological basis of:	- Musculoskeletal pain?
	- Other?
2) Should we investigate the anatomical and/or (neuro)physiological basis of chiropractic treatment effects and responses upon:	- Musculoskeletal pain?
	- Other?
**B. Biomechanics -** Should we investigate the biomechanics of:	- Spinal manipulative therapy
	- Different manipulative and manual techniques, as a basis for comparison
	- Other
**C. Diagnostic -** Should we investigate the following methods and techniques for the diagnosis of musculoskeletal pain:	- Imaging e.g. MRI, PET, CT, ultrasound
	- Postural and movement patterns
	- Thermal imaging and electromyography (EMG)
	- Eye movement patterns and visual perception
	- Other
**D. Theoretical concepts -** Should we investigate the theories and/or theoretical models of:	- The phases of spinal degeneration
	- The fixation/subluxation
	- Other
**III. Education**	
Should we examine:	- How student selection is conducted and how to select the best students
	- Curriculum design and implementation of curricula
	- Modes of delivery of postgraduate education
	- Philosophy of chiropractic care

**Table 4:** Results from phase 2, rounds 1 and 2

**Items that were thought to be important, reached consensus and removed from further voting**	**Items that were added following comments from the participants**	**Items that met consensus and were viewed to be important**
1. Cost-effectiveness of treatment	1. Effect of chiropractic treatment on:	1. Initiation and promotion of collaborative research efforts
2. Long-term effects of treatment	i. General health and well-being	2. Promotion of chiropractors to obtain their PhDs
	ii. Function and performance	3. Dose-response and frequency of treatment
3. Identification of sub-groups likely to respond to treatment	iii. Quality of life	4. Biological variables as predictors of outcome
	iv. Patient satisfaction	5. Effects and response of treatment on:
4. Effects and response of chiropractic treatment on musculoskeletal pain	v. Non-musculoskeletal conditions	i. Function and performance
5. Clinical findings as predictors of outcome	2. Comparison of the effects of chiropractic care with other professions	ii. Quality of life
6. Prevention of musculoskeletal pain in primary and secondary care	3. Effects of chiropractic care as part of a multi-modal package	6. Establishing clinical research networks throughout Europe
7. Investigate the anatomical and/or neurophysiological basis of chiropractic treatment on musculoskeletal pain	4. Effects of chiropractic treatment on the following specific populations:	7. Prognostic research on the clinical course of musculoskeletal pain
	i. Severely injured or disabled	8. Effects and response in the following specific populations:
	ii. Adults	i. Working population, including prevention in injured workers
	iii. Working population, including injured workers	ii. Geriatrics
	5. Examine:	iii. Adolescents
	i. Interaction between biological and psychosocial variables	9. Prevalence, incidence and prevention of musculoskeletal conditions in a working population
	ii. Patient expectations as predictors of outcome	
	iii. Role of imaging as predictor of outcome	
	6. Examine prevalence/incidence/prevention of musculoskeletal conditions in the following specific populations:	
	i. Adults	
	ii. Working population, including injured workers	
	7. Further explore the following:	
	i. Nature of practice/practice behavior	
	ii. Referral patterns of chiropractors to other professional groups	
	iii. Ethics of chiropractic practice	
	8. Examine the anatomical and/or neurophysiological basis of treatment on:	
	i. Disability and function	
	ii. Neurological processes	
	9. Investigate the biomechanics of normal and abnormal joint biomechanics	
	10. Investigate the role of fluoroscopy and functional imaging in diagnosing musculoskeletal pain	
	11. Examine the role of inter-professional learning within chiropractic education	
	12. Promote chiropractic PhDs in academic institutions throughout Europe	
	13. Establish clinical research networks throughout Europe	
	14. Initiate and promote collaborative research activity	

Note: Disagreement was identified for one item (i.e. theory of the subluxation).

Disagreement was identified for one item (i.e. ‘theory of the subluxation’) during the process, meaning that some (>30%) found this item important while others (>30%) found this unimportant, indicating clearly divergent ideas surrounding this theme.

No items were found to be unimportant or of uncertain importance.

### Prioritization of the items

In total, 19 of the 70 items reached consensus during the Delphi process, which were subsequently prioritized by the participants and are listed in hierarchal order in Table 5. Based on this rating, the top three items were: 1) cost-effectiveness/economic evaluations (34%), 2) identification of subgroups likely to respond to treatment (17%), and 3) initiation and promotion of collaborative research efforts (10%). Very few of the other items were viewed by the participants to be most important.

**Table 5:** Consensus items identified during the Delphi process ranked hierarchically in order of importance

**Rank**	**Important**		**Voted most important item (%)**^**1**^
1	Cost-effectiveness/economic evaluations		34
2	Identification of subgroups likely to respond to treatment		17
3	Initiation and promotion of collaborative research efforts		10
4	Promotion of chiropractors to obtain PhD’s		5
5		i. Dose response and frequency of treatment	2
		ii. Biopsychosocial variables as predictors of outcome	0
		iii. Anatomical &/or neurophysiological basis of chiropractic treatment on MSK pain	7
8	Effects and response of treatment on function and performance		0
9	Establishing clinical research networks throughout Europe		5
10	Prevention of MSK pain in primary and secondary care		7
11	Effects and response of treatment on quality of life		2
12	Effects and response of treatment on MSK pain		5
13	Clinical findings as predictors of outcome		0
14		i. Treatment and effects in adolescents	2
		ii. Prognostic research on the clinical course of MSK pain	0
16	Treatment and effects in a working population, prevention in injured workers		2
17		i. Long-term effects of treatment	0
		ii. Treatment and effects in geriatrics	0
19	Prevalence, incidence and prevention of MSK conditions in a working population		0

*Abbreviations:MSK* musculoskeletal.

^1^Percentage of the participants that voted the given consensus item as most important (i.e. number one priority).

Note: No items were found to be unimportant or of uncertain importance. Items that are indented received equal numbers of votes.

### Results from phase 3

Items that were presented during the 2013 annual European chiropractic forum were discussed amongst the project team directly following the meeting; however, this did not result in any modification to our list of items. It did, however, raise issues to be discussed. For example, one comment was raised that the lack of funding available was in direct conflict with the number one priority, namely, conducting an economic evaluation. In addition, a question was raised why the North American research agenda was not implemented on a greater scale, which we discuss further.

## Discussion

To our knowledge this is the first formal and systematic effort to propose a research agenda for the chiropractic profession in Europe. The Delphi technique used here was thought to be the method of choice for this type of research question, however, it is not without critique [11,12]. Although there are limitations to this study as with any study, great effort was taken to establish a transparent, pan-European chiropractic agenda. It is our hope, therefore, that these results will be used by both researchers and funding bodies alike. Although a previous list of European research priorities was established in 2011 via an iterative process and conducted among a limited number of chiropractic professionals, it was never published in a peer-reviewed journal.

There have been similar attempts in North America to establish research priorities for the chiropractic profession [5] or for the purpose of establishing a strategic plan to promote chiropractic research [2]. Other efforts have resulted in a research agenda aimed at improving patient care for common musculoskeletal problems through non-pharmacological therapies [6]. The aforementioned research agenda which examined non-pharmacological therapies followed an intensive workshop in the UK in 2007 and consisted of 30 researchers from a range of health professions experienced in clinical trials for musculoskeletal conditions in addition to two patient representatives [6]. Following that workshop, the results were presented at an international physical therapy symposium and the priorities were discussed with colleagues from other countries. Some of the priorities stemming from that process included the focus on implementation of research findings in clinical practice, development of research networks and inclusion of more innovative trial designs, such as stepped care. Three of the priorities identified during that process, namely cost-effectiveness, identification of subgroups and development of research networks, were also identified in our study suggesting that these are important items for chiropractic research as well. This is supported by the observation that there is a plethora of chiropractic literature, both in peer-reviewed and professional journals, that have discussed the importance of subgroups, and to a lesser extent, cost-effectiveness.

Our list of priorities is a mix of research themes or topics and strategies; however, that is not unlike the previously discussed research agenda initiated in the UK [6]. For example, cost-effectiveness and identification of subgroups are specific areas of research, while the initiation and promotion of collaborative research efforts and the promotion of chiropractic PhDs are strategies to advance the academic integration of the profession. We can only conclude from this that there appears to be a need to promote both efforts within the profession and that apparently researchers with a background in chiropractic are eager to engage in multidisciplinary research efforts.

If these priorities are to be implemented, it would benefit from an organized approach. In this regard, we view this survey as a first step in promoting a more unified approach towards European chiropractic research. However, we have concerns that this survey will have limited impact if we are to draw a parallel with a similar process conducted in 1997 by North American colleagues [3]. At that time, six general recommendations for chiropractic care in North America were made: 1) determine barriers to usage; 2) develop models to explain usage; 3) determine cost-effectiveness of different chiropractic procedures; 4) develop valid measures; 5) develop predictors of quality of care; and, 6) examine satisfaction with chiropractic services. Following that initial publication, an update was conducted in 2006, and the authors concluded that none of the items proposed in 1997 had been adequately addressed, although all the items were deemed important and relevant [5]. The reasons why the agenda was not implemented on a greater scale remain unclear, but the authors seem to suggest that the lack of an ‘…organized effort on the part of chiropractic institutions and organizations…’ might be an important reason.

Other concerns regard the desirability or feasibility of implementation of the proposed agenda. Most notably, there are fundamental differences in culture, healthcare systems and policies of reimbursement as well as the position of chiropractic within each of the European countries. This will certainly limit the possibilities of collaborative efforts even if they are desirable. In addition, the number one priority, an economic evaluation, is best addressed via a randomized study design. Needless to say, trials are expensive and funding for chiropractic research is limited.

In an attempt to address some of these issues, a number of steps have been taken to promote knowledge transfer and implementation. For example, an executive summary of this study has been drafted and distributed to members of the ECU Executive Council, which can influence policy making. The results have also been presented to the ECU Executive and General Councils and at the annual ECU/EAC Researchers’ Day and ECU Convention. This provided opportunities for discussion and a forum for politicians, researchers and clinicians to become familiar with these priorities. In hindsight, it might have been more appropriate to conduct a focus group rather than discussion following a plenary presentation, which might have facilitated the discussion process better.

### Strengths and limitations

An important strength of this study includes participation and ranking of the items by researchers prominent in the European chiropractic profession and thus, well familiar with the literature. Potential sources of bias include selection bias and over-representation from UK and Danish institutions. Furthermore, other stakeholders could have been involved from the beginning of this process which may have resulted in a more nuanced agenda or different items. On the other hand, the participants of this study have wide-ranging knowledge of the literature, in addition to extensive experience in clinical chiropractic practice. Other limitations include the possibility that individual participants might have recommended and/or prioritized their personal areas of research rather than indicate future items of interest for the profession. In addition, the inclusion of comments from the participants during the Delphi process was based upon interpretation of these comments by the project team; therefore, the decision to include an item or not might be considered subjective. This should not be considered a weakness inherent to this project alone, but a criticism of the Delphi process, in general [8,11]. Finally, our focus was on establishing a list of priorities for the chiropractic profession in Europe. While other research is necessary, certainly in primary care, other proposed items which were not unique to chiropractic care, such as understanding aetiological factors in specific populations with musculoskeletal conditions or better understanding the clinical course of musculoskeletal conditions, did not reach consensus, and thus, are not included here.

## Conclusions

This is the first formal and systematic attempt to establish a research agenda for the chiropractic profession in Europe. The top three items identified during this process were: 1) cost-effectiveness/economic evaluations, 2) identification of subgroups likely to respond to treatment, and 3) initiation and promotion of collaborative research efforts. Future discussion and studies will be necessary to determine whether the themes identified in this survey should be broadly implemented.

## Abbreviations

ECU: European Chiropractors’ Union; EAC: European Academy of Chiropractic.

## Competing interests

All authors possess a chiropractic degree and/or work in chiropractic institutions. Funding was received from the European Chiropractic Union (project no. A12.04).

## Authors' contributions

Conception and design: all authors. Analysis and interpretation of the data: SMR, AW, JH. Drafting of the review: all authors. Critical revision of the article for important intellectual content: all authors. Final approval of the article: all authors. Statistical expertise: none necessary beyond the capacity of the authors.

## Supplementary Material

Additional file 1 **Appendix 1.** Open-ended data collection tool. **Appendix 2.** Questionnaire at phase 2, Round 1. **Appendix 3.** Questionnaire at phase 2, Round 2. **Appendix 4.** Comments from the participants at phase 2, Rounds 1 and 2. **Appendix 5.** Questionnaire at phase 2, Round 3. Click here for file
