# Supplementary material for: The first research agenda for the chiropractic profession in Europe
Source: Chiropr Man Therap. 2014 Feb 10;22:9. doi: 10.1186/2045-709X-22-9 (PMC3942182; doi:10.1186/2045-709X-22-9)
Supplement: Additional file 1 — Appendix 1. Open-ended data collection tool. Appendix 2. Questionnaire at phase 2, Round 1. Appendix 3. Questionnaire at phase 2, Round 2. Appendix 4. Comments from the participants at phase 2, Rounds 1 and 2. Appendix 5. Questionnaire at phase 2, Round 3. [file 2045-709X-22-9-S1.pdf]

Appendix 1. Invitation letter and data collection tool for the first round (identification of themes).

Invitation to participate in *Research Priorities for the Chiropractic Profession in Europe*

Research involving chiropractors is evolving and expanding in several countries and the number of published research papers from chiropractors is rapidly increasing. Most of these projects and papers include chiropractors as part of multidisciplinary research groups, however it is not clear whether this is because of a conscious strategy towards integration or because chiropractors do not have the ability, possibility or desire to carry out their own ”chiropractic” research agenda.

At the ECU Research Day meeting in Zurich this past spring, it was decided that we needed to establish a vision for chiropractic research in Europe for the coming 5 to 10 years. In order to fulfill this goal, we want to aim towards establishing a set of research priorities for the chiropractic profession in Europe. The process involves the following four steps:

1. Identification of themes
Firstly, we will ask researchers with a chiropractic background, and who have attended the ECU Research Day, to name some specific themes that are feasible as research priorities for the chiropractic profession in Europe. The Steering Group, consisting of Alexandra Webb, Jenni Bolton, Sidney Rubinstein and Jan Hartvigsen, will then organize these themes into categories.

2. Delphi process
Secondly, the list of categories and themes will be circulated and you will then be asked to prioritize them. We envision that two or three rounds will be necessary.

3. Workshop
Thirdly, a workshop involving clinicians, politicians and researchers will be held at the 2013 ECU conference. The aim of this workshop is to encourage input and comments from participants and hopefully agree on a list of priorities.

4. Publication
Lastly, it is our goal to put the list of research priorities into context and publish this as a position paper in the Journal of Chiropractic and Manual Therapies. The Steering Group will co-author this paper. All other participants in step one and two above will be acknowledged.

We kindly invite you to join this project by participating in steps one and two. Attached please find the form for step one.

Please return the form to Jytte Johannesen at the Nordic Institute of Chiropractic and Clinical Biomechanics October 15: J.johannesen@nikkb.dk

If you have any questions, please do not hesitate to contact any or all members of the Steering Group or Jytte Johannesen, who can forward your comments.

We thank you in advance.

With best wishes,
Alexander Webb
Jennifer Bolton
Sidney Rubinstein
Jan Hartvigsen

Research Priorities for the Chiropractic Profession in Europe

In the boxes below, please write as specific as possible the research topics you consider important. In this initial round you do not need to place the topics in any particular order or prioritize them. Feel free to suggest as many topics as you want.<br><br>In subsequent rounds we will ask you to rate the importance of all the topics that have come forward in order to arrive at a list of research priorities for the chiropractic profession in Europe.
**Epidemiological research:**
**Clinical research:**
**Basic science/mechanistic research:**
**Other research:**
**Your name and email:**

Thank you for your time and assistance.

Appendix 2. Phase 2, Round 1

The items have been organized into the following areas: 1) epidemiological and clinical research, 2) basic science research, and 3) education, and in the following screens we ask you to rate the importance of each item, ranging from "extremely unimportant" to "extremely important".

You "start" the questionnaire by clicking the right arrow at the bottom of this screen.
In the next screens you use the right and left arrows at the bottom to move forwards and backwards between the screens e.g. to review or change answers.

SOCIODEMOGRAPHIC DATA

1. What is your age?

____

2. What is your gender?

(1) ❑ Male

(2) ❑ Female

3. What is the highest academic degree which you have obtained? (please give the full name of the degree and the name of the awarding institution)

____

____

4. Do you have a degree in chiropractic?

(1) ❑ Yes. Go to question 5

(2) ❑ No. Go to question 6

5. If you have a chiropractic degree:

A. What college/university did you attend? ____

B. What year did you graduate? ____

6. Do you primarily work in:

(1) ❑ Clinical practice

(2) ❑ Educational institution - teaching and/or research

(3) ❑ Administration

(4) ❑ Other. Please specify __________

7. In what country do you reside?

____

I. EPIDEMIOLOGICAL AND CLINICAL RESEARCH

A. Effects of treatment - The following refer specifically to the treatment effects of chiropractic care.

1. Should more research be conducted in the following areas?

	1 Extremely unimportant	2	3	4	5	6	7	8	9 Extremely important
a. Cost-effectiveness (i.e. conduct economic evaluations)	(1) ❑	(2) ❑	(3) ❑	(4) ❑	(5) ❑	(6) ❑	(7) ❑	(8) ❑	(9) ❑

	1 Extremely unimportant	2	3	4	5	6	7	8	9 Extremely important
b. Maintenance care	(1) ❑	(2) ❑	(3) ❑	(4) ❑	(5) ❑	(6) ❑	(7) ❑	(8) ❑	(9) ❑
c. Short-term effects (<3 months)	(1) ❑	(2) ❑	(3) ❑	(4) ❑	(5) ❑	(6) ❑	(7) ❑	(8) ❑	(9) ❑
d. Long-term effects (>9 months)	(1) ❑	(2) ❑	(3) ❑	(4) ❑	(5) ❑	(6) ❑	(7) ❑	(8) ❑	(9) ❑
e. Sub-groups likely to respond to care	(1) ❑	(2) ❑	(3) ❑	(4) ❑	(5) ❑	(6) ❑	(7) ❑	(8) ❑	(9) ❑
f. Safety/adverse events	(1) ❑	(2) ❑	(3) ❑	(4) ❑	(5) ❑	(6) ❑	(7) ❑	(8) ❑	(9) ❑
g. Dose-response and frequency of treatment	(1) ❑	(2) ❑	(3) ❑	(4) ❑	(5) ❑	(6) ❑	(7) ❑	(8) ❑	(9) ❑
h. Comparison of different chiropractic techniques	(1) ❑	(2) ❑	(3) ❑	(4) ❑	(5) ❑	(6) ❑	(7) ❑	(8) ❑	(9) ❑
i. Other	(1) ❑	(2) ❑	(3) ❑	(4) ❑	(5) ❑	(6) ❑	(7) ❑	(8) ❑	(9) ❑

1.i. If you have marked the item "Other" as a 5 or higher in importance, please specify that here.

____

2. Should more research be conducted on chiropractic treatment effects and responses upon:

	1 Extremely unimportant	2	3	4	5	6	7	8	9 Extremely important
a. Musculoskeletal pain?	(1) ❑	(2) ❑	(3) ❑	(4) ❑	(5) ❑	(6) ❑	(7) ❑	(8) ❑	(9) ❑
b. Other?	(1) ❑	(2) ❑	(3) ❑	(4) ❑	(5) ❑	(6) ❑	(7) ❑	(8) ❑	(9) ❑

2.b. If you have marked the item "Other" as a 5 or higher in importance, please specify that here.

____

3. Should more research be conducted on chiropractic treatment effects and responses in any of the following specific populations?

	1 Extremely unimportant	2	3	4	5	6	7	8	9 Extremely important
a. Infants and babies	(1) ❑	(2) ❑	(3) ❑	(4) ❑	(5) ❑	(6) ❑	(7) ❑	(8) ❑	(9) ❑
b. Pre-school and children	(1) ❑	(2) ❑	(3) ❑	(4) ❑	(5) ❑	(6) ❑	(7) ❑	(8) ❑	(9) ❑
c. Adolescents	(1) ❑	(2) ❑	(3) ❑	(4) ❑	(5) ❑	(6) ❑	(7) ❑	(8) ❑	(9) ❑
d. Geriatrics	(1) ❑	(2) ❑	(3) ❑	(4) ❑	(5) ❑	(6) ❑	(7) ❑	(8) ❑	(9) ❑
e. Pregnant women	(1) ❑	(2) ❑	(3) ❑	(4) ❑	(5) ❑	(6) ❑	(7) ❑	(8) ❑	(9) ❑

	1 Extremely unimportant	2	3	4	5	6	7	8	9 Extremely important
f. Athletes	(1) ❑	(2) ❑	(3) ❑	(4) ❑	(5) ❑	(6) ❑	(7) ❑	(8) ❑	(9) ❑
g. Other	(1) ❑	(2) ❑	(3) ❑	(4) ❑	(5) ❑	(6) ❑	(7) ❑	(8) ❑	(9) ❑

3.g. If you have marked the item "Other" as a 5 or higher in importance, please specify that here.

____

If there is something missing from the lists regarding effects of treatment, please feel free to add your comments.

____

____

____

____

____

____

B. Prognostic research

1. Should more prognostic research be conducted on the clinical course of musculoskeletal pain (for the identification of subgroups) in:

	1 Extremely unimportant	2	3	4	5	6	7	8	9 Extremely important
a. a chiropractic population?	(1) ❑	(2) ❑	(3) ❑	(4) ❑	(5) ❑	(6) ❑	(7) ❑	(8) ❑	(9) ❑
b. population treated by other health care practitioners?	(1) ❑	(2) ❑	(3) ❑	(4) ❑	(5) ❑	(6) ❑	(7) ❑	(8) ❑	(9) ❑

2. Should we examine any of the following specific factors as predictors of outcome with chiropractic treatment?

	1 Extremely unimportant	2	3	4	5	6	7	8	9 Extremely important
a. Psychosocial factors	(1) ❑	(2) ❑	(3) ❑	(4) ❑	(5) ❑	(6) ❑	(7) ❑	(8) ❑	(9) ❑
b. Clinical findings	(1) ❑	(2) ❑	(3) ❑	(4) ❑	(5) ❑	(6) ❑	(7) ❑	(8) ❑	(9) ❑
c. Other	(1) ❑	(2) ❑	(3) ❑	(4) ❑	(5) ❑	(6) ❑	(7) ❑	(8) ❑	(9) ❑

2.c. If you have marked the item "Other" as a 5 or higher in importance, please specify that here.

____

If there is something missing from the lists regarding prognostic research, please feel free to add your comments.

____

____

____

____

____

____

C. Prevalence/incidence/prevention/population studies of musculoskeletal conditions

1. Should we examine prevalence/incidence/prevention of musculoskeletal conditions in the following specific patient populations?

	1 Extremely unimportant	2	3	4	5	6	7	8	9 Extremely important
a. Infants and babies	(1) ❑	(2) ❑	(3) ❑	(4) ❑	(5) ❑	(6) ❑	(7) ❑	(8) ❑	(9) ❑
b. Pre-school and children	(1) ❑	(2) ❑	(3) ❑	(4) ❑	(5) ❑	(6) ❑	(7) ❑	(8) ❑	(9) ❑
c. Adolescents	(1) ❑	(2) ❑	(3) ❑	(4) ❑	(5) ❑	(6) ❑	(7) ❑	(8) ❑	(9) ❑
d. Geriatrics	(1) ❑	(2) ❑	(3) ❑	(4) ❑	(5) ❑	(6) ❑	(7) ❑	(8) ❑	(9) ❑
e. Pregnant women	(1) ❑	(2) ❑	(3) ❑	(4) ❑	(5) ❑	(6) ❑	(7) ❑	(8) ❑	(9) ❑
f. Athletes	(1) ❑	(2) ❑	(3) ❑	(4) ❑	(5) ❑	(6) ❑	(7) ❑	(8) ❑	(9) ❑
g. Other	(1) ❑	(2) ❑	(3) ❑	(4) ❑	(5) ❑	(6) ❑	(7) ❑	(8) ❑	(9) ❑

1.g. If you have marked the item "Other" as a 5 or higher in importance, please specify that here.

____

2. Should we examine the following specific topics?

	1 Extremely unimportant	2	3	4	5	6	7	8	9 Extremely important

	1 Extremely unimportant	2	3	4	5	6	7	8	9 Extremely important
a. Determinants of work absenteeism for musculoskeletal conditions	(1) ❑	(2) ❑	(3) ❑	(4) ❑	(5) ❑	(6) ❑	(7) ❑	(8) ❑	(9) ❑
b. Descriptive studies on clinics, chiropractors and patients in all ECU member countries	(1) ❑	(2) ❑	(3) ❑	(4) ❑	(5) ❑	(6) ❑	(7) ❑	(8) ❑	(9) ❑
c. Risk factors for incidence of musculoskeletal pain	(1) ❑	(2) ❑	(3) ❑	(4) ❑	(5) ❑	(6) ❑	(7) ❑	(8) ❑	(9) ❑
d. Prevention of musculoskeletal pain in primary and secondary care	(1) ❑	(2) ❑	(3) ❑	(4) ❑	(5) ❑	(6) ❑	(7) ❑	(8) ❑	(9) ❑
e. Other	(1) ❑	(2) ❑	(3) ❑	(4) ❑	(5) ❑	(6) ❑	(7) ❑	(8) ❑	(9) ❑

2.e. If you have marked the item "Other" as a 5 or higher in importance, please specify that here.

____

If there is something missing from the lists regarding prevalence/incidence/prevention/population studies of musculoskeletal conditions, please feel free to add your comments.

____

____

____

____

____

____

D. Issues of chiropractic practice

1. Should we examine:

	1 Extremely unimportant	2	3	4	5	6	7	8	9 Extremely important
a. the clinician-patient relationship?	(1) ❑	(2) ❑	(3) ❑	(4) ❑	(5) ❑	(6) ❑	(7) ❑	(8) ❑	(9) ❑
b. who are the care seekers and what triggers their care seeking?	(1) ❑	(2) ❑	(3) ❑	(4) ❑	(5) ❑	(6) ❑	(7) ❑	(8) ❑	(9) ❑
c. other?	(1) ❑	(2) ❑	(3) ❑	(4) ❑	(5) ❑	(6) ❑	(7) ❑	(8) ❑	(9) ❑

1.c. If you have marked the item "other" as a 5 or higher in importance, please specify that here.

____

If there is something missing from the list regarding issues of chiropractic practice, please feel free to add your comments.

____

____

____

____

____

____

II. BASIC SCIENCE RESEARCH

A. Anatomy and physiology

1. Should we investigate the anatomical and/or (neuro)physiological basis of:

	1 Extremely unimportant	2	3	4	5	6	7	8	9 Extremely important
a. musculoskeletal pain?	(1) ❑	(2) ❑	(3) ❑	(4) ❑	(5) ❑	(6) ❑	(7) ❑	(8) ❑	(9) ❑
b. other?	(1) ❑	(2) ❑	(3) ❑	(4) ❑	(5) ❑	(6) ❑	(7) ❑	(8) ❑	(9) ❑

1.b. If you have marked the item "other" as a 5 or higher in importance, please specify that here.

____

2. Should we investigate the anatomical and/or (neuro)physiological basis of chiropractic treatment effects and responses upon:

	1 Extremely unimportant	2	3	4	5	6	7	8	9 Extremely important
a. musculoskeletal pain?	(1) ❑	(2) ❑	(3) ❑	(4) ❑	(5) ❑	(6) ❑	(7) ❑	(8) ❑	(9) ❑
b. other?	(1) ❑	(2) ❑	(3) ❑	(4) ❑	(5) ❑	(6) ❑	(7) ❑	(8) ❑	(9) ❑

2.b. If you have marked the item "other" as a 5 or higher in importance, please specify that here.

____

If there is something missing from the lists regarding anatomy and physiology, please feel free to add your comments.

____

____

____

____

____

____

B. Biomechanics

1. Should we investigate the biomechanics of:

1 Extremely unimportant	2	3	4	5	6	7	8	9 Extremely important

	1 Extremely unimportant	2	3	4	5	6	7	8	9 Extremely important
a. spinal manipulative therapy?	(1) ❑	(2) ❑	(3) ❑	(4) ❑	(5) ❑	(6) ❑	(7) ❑	(8) ❑	(9) ❑
b. different manipulative and manual techniques, as a basis for comparison?	(1) ❑	(2) ❑	(3) ❑	(4) ❑	(5) ❑	(6) ❑	(7) ❑	(8) ❑	(9) ❑
c. other?	(1) ❑	(2) ❑	(3) ❑	(4) ❑	(5) ❑	(6) ❑	(7) ❑	(8) ❑	(9) ❑

1.c. If you have marked the item "other" as a 5 or higher in importance, please specify that here.

____

If there is something missing from the list regarding biomechanics, please feel free to add your comments.

____

____

____

____

____

____

C. Diagnostic

1. Should we investigate the following methods and techniques for the diagnosis of musculoskeletal pain:

	1 Extremely unimportant	2	3	4	5	6	7	8	9 Extremely important
a. imaging e.g. MRI, PET, CT, ultrasound?	(1) ❑	(2) ❑	(3) ❑	(4) ❑	(5) ❑	(6) ❑	(7) ❑	(8) ❑	(9) ❑
b. postural and movement patterns?	(1) ❑	(2) ❑	(3) ❑	(4) ❑	(5) ❑	(6) ❑	(7) ❑	(8) ❑	(9) ❑
c. thermal imaging and electromyography (EMG)?	(1) ❑	(2) ❑	(3) ❑	(4) ❑	(5) ❑	(6) ❑	(7) ❑	(8) ❑	(9) ❑
d. eye movement patterns and visual perception?	(1) ❑	(2) ❑	(3) ❑	(4) ❑	(5) ❑	(6) ❑	(7) ❑	(8) ❑	(9) ❑
e. other?	(1) ❑	(2) ❑	(3) ❑	(4) ❑	(5) ❑	(6) ❑	(7) ❑	(8) ❑	(9) ❑

1.e. If you have marked the item "other" as a 5 or higher in importance, please specify that here.

____

If there is something missing from the list regarding diagnostic, please feel free to add your comments.

____

____

____

____

____

____

D. Theoretical concepts

1. Should we investigate the theories and/or theoretical models of:

	1 Extremely unimportant	2	3	4	5	6	7	8	9 Extremely important
a. the phases of spinal degeneration?	(1) ❑	(2) ❑	(3) ❑	(4) ❑	(5) ❑	(6) ❑	(7) ❑	(8) ❑	(9) ❑

	1 Extremely unimportant	2	3	4	5	6	7	8	9 Extremely important
b. the fixation/subluxation?	(1) ❑	(2) ❑	(3) ❑	(4) ❑	(5) ❑	(6) ❑	(7) ❑	(8) ❑	(9) ❑
c. other?	(1) ❑	(2) ❑	(3) ❑	(4) ❑	(5) ❑	(6) ❑	(7) ❑	(8) ❑	(9) ❑

1.c. If you have marked the item "other" as a 5 or higher in importance, please specify that here.

____

If there is something missing from the list regarding theoretical concepts, please feel free to add your comments.

____

____

____

____

____

____

III. EDUCATION

1. Should we examine:

	1 Extremely unimportant	2	3	4	5	6	7	8	9 Extremely important
a. how student selection is conducted and how to select the best students?	(1) ❑	(2) ❑	(3) ❑	(4) ❑	(5) ❑	(6) ❑	(7) ❑	(8) ❑	(9) ❑
b. curriculum design and implementation of curricula?	(1) ❑	(2) ❑	(3) ❑	(4) ❑	(5) ❑	(6) ❑	(7) ❑	(8) ❑	(9) ❑
c. modes of delivery of postgraduate education?	(1) ❑	(2) ❑	(3) ❑	(4) ❑	(5) ❑	(6) ❑	(7) ❑	(8) ❑	(9) ❑
d. philosophy of chiropractic care?	(1) ❑	(2) ❑	(3) ❑	(4) ❑	(5) ❑	(6) ❑	(7) ❑	(8) ❑	(9) ❑

If there is something missing from the list regarding education, please feel free to add your comments.

____

____

____

____

____

____

We are also interested in your ideas as to how we can actually implement research into these topics. All comments are welcome.

____

____

____

____

____

____

Thank you very much for your participation. We will keep you informed on the progress of this study and look forward to seeing you in May in Amsterdam.

If you have answered all the questions and do not want to change answers, please click the X below to complete and send the questionnaire.

Appendix 3. Phase 2, Round 2

The items have been organized into the following areas: I. Epidemiological and clinical research, II. Basic science research, III. Education and IV. Other. In the following screens we ask you to rate the importance of each item, ranging from "extremely unimportant" to "extremely important".

You "start" the questionnaire by clicking the right arrow at the bottom of this screen.
In the next screens you use the right and left arrows at the bottom to move forwards and backwards between the screens e.g. to review or change answers.

Already approved items

For your information the following items have already reached consensus, meaning they have been found by the majority of the group to warrant further investigation.

I. EPIDEMIOLOGICAL AND CLINICAL RESEARCH

Effects of chiropractic treatment

Cost-effectiveness

Long-term effects of treatment

Identification of subgroups likely to respond to treatment

Effects of chiropractic treatment on musculoskeletal pain

Prognostic research

Clinical findings as predictors of outcome

Prevention Prevention of musculoskeletal pain in primary and secondary care

II. BASIC SCIENCE RESEARCH

Anatomy and physiology

Investigate the anatomical and/or neurophysiological basis of chiropractic treatment on musculoskeletal pain.

These items have been removed for further voting. However, you are welcome to provide comments below.

Please provide any comments here

____

____

____

____

____

____

I. EPIDEMIOLOGICAL AND CLINICAL RESEARCH

A. Effects of treatment - The following refer specifically to the treatment effects of chiropractic care.

1. Should more research be conducted in the following areas?

	1 Extremely unimportant	2	3	4	5	6	7	8	9 Extremely important
a. Maintenance care	(1) ❑	(2) ❑	(3) ❑	(4) ❑	(5) ❑	(6) ❑	(7) ❑	(8) ❑	(9) ❑
b. Short-term effects (<3 months)	(1) ❑	(2) ❑	(3) ❑	(4) ❑	(5) ❑	(6) ❑	(7) ❑	(8) ❑	(9) ❑
c. Safety/adverse events	(1) ❑	(2) ❑	(3) ❑	(4) ❑	(5) ❑	(6) ❑	(7) ❑	(8) ❑	(9) ❑
d. Dose-response and frequency of treatment	(1) ❑	(2) ❑	(3) ❑	(4) ❑	(5) ❑	(6) ❑	(7) ❑	(8) ❑	(9) ❑
e. Comparison of different chiropractic techniques	(1) ❑	(2) ❑	(3) ❑	(4) ❑	(5) ❑	(6) ❑	(7) ❑	(8) ❑	(9) ❑

	1 Extremely unimportant	2	3	4	5	6	7	8	9 Extremely important
f. Comparison of the effects of chiropractic care with other professions	(1) ❑	(2) ❑	(3) ❑	(4) ❑	(5) ❑	(6) ❑	(7) ❑	(8) ❑	(9) ❑
g. Effects of chiropractic care as part of a multi-modal package	(1) ❑	(2) ❑	(3) ❑	(4) ❑	(5) ❑	(6) ❑	(7) ❑	(8) ❑	(9) ❑

2. Should more research be conducted on chiropractic treatment effects and responses upon:

	1 Extremely unimportant	2	3	4	5	6	7	8	9 Extremely important
a. "Wellness"; general health and well-being	(1) ❑	(2) ❑	(3) ❑	(4) ❑	(5) ❑	(6) ❑	(7) ❑	(8) ❑	(9) ❑
b. Function and performance	(1) ❑	(2) ❑	(3) ❑	(4) ❑	(5) ❑	(6) ❑	(7) ❑	(8) ❑	(9) ❑
c. Quality of life	(1) ❑	(2) ❑	(3) ❑	(4) ❑	(5) ❑	(6) ❑	(7) ❑	(8) ❑	(9) ❑
d. Patient satisfaction	(1) ❑	(2) ❑	(3) ❑	(4) ❑	(5) ❑	(6) ❑	(7) ❑	(8) ❑	(9) ❑
e. Non-musculoskeletal conditions	(1) ❑	(2) ❑	(3) ❑	(4) ❑	(5) ❑	(6) ❑	(7) ❑	(8) ❑	(9) ❑

3. Should more research be conducted on chiropractic treatment effects and responses in any of the following specific populations?

	1 Extremely unimportant	2	3	4	5	6	7	8	9 Extremely important
a. Infants and babies	(1) ❑	(2) ❑	(3) ❑	(4) ❑	(5) ❑	(6) ❑	(7) ❑	(8) ❑	(9) ❑
b. Pre-school and children	(1) ❑	(2) ❑	(3) ❑	(4) ❑	(5) ❑	(6) ❑	(7) ❑	(8) ❑	(9) ❑
c. Adolescents	(1) ❑	(2) ❑	(3) ❑	(4) ❑	(5) ❑	(6) ❑	(7) ❑	(8) ❑	(9) ❑
d. Adults	(1) ❑	(2) ❑	(3) ❑	(4) ❑	(5) ❑	(6) ❑	(7) ❑	(8) ❑	(9) ❑
e. Geriatrics	(1) ❑	(2) ❑	(3) ❑	(4) ❑	(5) ❑	(6) ❑	(7) ❑	(8) ❑	(9) ❑
f. Pregnant women	(1) ❑	(2) ❑	(3) ❑	(4) ❑	(5) ❑	(6) ❑	(7) ❑	(8) ❑	(9) ❑
g. Athletes	(1) ❑	(2) ❑	(3) ❑	(4) ❑	(5) ❑	(6) ❑	(7) ❑	(8) ❑	(9) ❑
h. Severely injured or disabled persons	(1) ❑	(2) ❑	(3) ❑	(4) ❑	(5) ❑	(6) ❑	(7) ❑	(8) ❑	(9) ❑
i. Working populations, work disability prevention and injured workers	(1) ❑	(2) ❑	(3) ❑	(4) ❑	(5) ❑	(6) ❑	(7) ❑	(8) ❑	(9) ❑

If there is something missing from the lists regarding effects of treatment, please feel free to add your comments.

I. EPIDEMIOLOGICAL AND CLINICAL RESEARCH

B. Prognostic research

1. Should more prognostic research be conducted on the clinical course of musculoskeletal pain (for the identification of subgroups) in:

	1 Extremely unimportant	2	3	4	5	6	7	8	9 Extremely important
a. A chiropractic population?	(1) ❑	(2) ❑	(3) ❑	(4) ❑	(5) ❑	(6) ❑	(7) ❑	(8) ❑	(9) ❑
b. Population treated by other health care practitioners?	(1) ❑	(2) ❑	(3) ❑	(4) ❑	(5) ❑	(6) ❑	(7) ❑	(8) ❑	(9) ❑

2. Should we examine any of the following specific factors as predictors of outcome with chiropractic treatment?

	1 Extremely unimportant	2	3	4	5	6	7	8	9 Extremely important
a. Psychosocial factors	(1) ❑	(2) ❑	(3) ❑	(4) ❑	(5) ❑	(6) ❑	(7) ❑	(8) ❑	(9) ❑
b. Interaction between biological and psychosocial variables	(1) ❑	(2) ❑	(3) ❑	(4) ❑	(5) ❑	(6) ❑	(7) ❑	(8) ❑	(9) ❑
c. Patient expectations	(1) ❑	(2) ❑	(3) ❑	(4) ❑	(5) ❑	(6) ❑	(7) ❑	(8) ❑	(9) ❑
d. Imaging (radiography, MRI)	(1) ❑	(2) ❑	(3) ❑	(4) ❑	(5) ❑	(6) ❑	(7) ❑	(8) ❑	(9) ❑

If there is something missing from the lists regarding prognostic research, please feel free to add your comments.

____

____

____

____

____

____

I. EPIDEMIOLOGICAL AND CLINICAL RESEARCH

C. Prevalence/incidence/prevention/population studies of musculoskeletal conditions

1. Should we examine prevalence/incidence/prevention of musculoskeletal conditions in the following specific patient populations?

	1 Extremely unimportant	2	3	4	5	6	7	8	9 Extremely important
a. Infants and babies	(1) ❑	(2) ❑	(3) ❑	(4) ❑	(5) ❑	(6) ❑	(7) ❑	(8) ❑	(9) ❑
b. Pre-school and children	(1) ❑	(2) ❑	(3) ❑	(4) ❑	(5) ❑	(6) ❑	(7) ❑	(8) ❑	(9) ❑
c. Adolescents	(1) ❑	(2) ❑	(3) ❑	(4) ❑	(5) ❑	(6) ❑	(7) ❑	(8) ❑	(9) ❑
d. Adults	(1) ❑	(2) ❑	(3) ❑	(4) ❑	(5) ❑	(6) ❑	(7) ❑	(8) ❑	(9) ❑
e. Geriatrics	(1) ❑	(2) ❑	(3) ❑	(4) ❑	(5) ❑	(6) ❑	(7) ❑	(8) ❑	(9) ❑
f. Pregnant women	(1) ❑	(2) ❑	(3) ❑	(4) ❑	(5) ❑	(6) ❑	(7) ❑	(8) ❑	(9) ❑
g. Athletes	(1) ❑	(2) ❑	(3) ❑	(4) ❑	(5) ❑	(6) ❑	(7) ❑	(8) ❑	(9) ❑
h. Severely injured or disabled persons	(1) ❑	(2) ❑	(3) ❑	(4) ❑	(5) ❑	(6) ❑	(7) ❑	(8) ❑	(9) ❑
i. Working populations, work disability prevention and injured workers	(1) ❑	(2) ❑	(3) ❑	(4) ❑	(5) ❑	(6) ❑	(7) ❑	(8) ❑	(9) ❑

2. Should we examine the following specific topics?

	1 Extremely unimportant	2	3	4	5	6	7	8	9 Extremely important
a. Determinants of work absenteeism for musculoskeletal conditions	(1) ❑	(2) ❑	(3) ❑	(4) ❑	(5) ❑	(6) ❑	(7) ❑	(8) ❑	(9) ❑
b. Descriptive studies on clinics, chiropractors and patients in all ECU member countries	(1) ❑	(2) ❑	(3) ❑	(4) ❑	(5) ❑	(6) ❑	(7) ❑	(8) ❑	(9) ❑
c. Risk factors for incidence of musculoskeletal pain	(1) ❑	(2) ❑	(3) ❑	(4) ❑	(5) ❑	(6) ❑	(7) ❑	(8) ❑	(9) ❑

If there is something missing from the lists regarding prevalence/incidence/prevention/population studies of musculoskeletal conditions, please feel free to add your comments.

____

____

____

____

____

____

I. EPIDEMIOLOGICAL AND CLINICAL RESEARCH

D. Issues of chiropractic practice

1. Should we examine:

	1 Extremely unimportant	2	3	4	5	6	7	8	9 Extremely important
a. The clinician-patient relationship?	(1) ❑	(2) ❑	(3) ❑	(4) ❑	(5) ❑	(6) ❑	(7) ❑	(8) ❑	(9) ❑
b. Who are the care seekers and what triggers their care seeking?	(1) ❑	(2) ❑	(3) ❑	(4) ❑	(5) ❑	(6) ❑	(7) ❑	(8) ❑	(9) ❑
c. Nature of practice / practice behaviour?	(1) ❑	(2) ❑	(3) ❑	(4) ❑	(5) ❑	(6) ❑	(7) ❑	(8) ❑	(9) ❑
d. Referral patterns of chiropractors to other professional groups?	(1) ❑	(2) ❑	(3) ❑	(4) ❑	(5) ❑	(6) ❑	(7) ❑	(8) ❑	(9) ❑
e. Ethics of chiropractic practice?	(1) ❑	(2) ❑	(3) ❑	(4) ❑	(5) ❑	(6) ❑	(7) ❑	(8) ❑	(9) ❑

If there is something missing from the list regarding issues of chiropractic practice, please feel free to add your comments.

____

____

____

____

____

____

II. BASIC SCIENCE RESEARCH

A. Anatomy and physiology

1. Should we investigate the anatomical and/or (neuro)physiological basis of:

	1 Extremely unimportant	2	3	4	5	6	7	8	9 Extremely important
a. Musculoskeletal pain?	(1) ❑	(2) ❑	(3) ❑	(4) ❑	(5) ❑	(6) ❑	(7) ❑	(8) ❑	(9) ❑

	1 Extremely unimportant	2	3	4	5	6	7	8	9 Extremely important
b. Disability and function?	(1) ❑	(2) ❑	(3) ❑	(4) ❑	(5) ❑	(6) ❑	(7) ❑	(8) ❑	(9) ❑

2. Should we investigate the anatomical and/or (neuro)physiological basis of chiropractic treatment effects and responses upon:

	1 Extremely unimportant	2	3	4	5	6	7	8	9 Extremely important
a. Disability and function?	(1) ❑	(2) ❑	(3) ❑	(4) ❑	(5) ❑	(6) ❑	(7) ❑	(8) ❑	(9) ❑
b. Neurological processes?	(1) ❑	(2) ❑	(3) ❑	(4) ❑	(5) ❑	(6) ❑	(7) ❑	(8) ❑	(9) ❑

If there is something missing from the lists regarding anatomy and physiology, please feel free to add your comments.

____

____

____

____

II. BASIC SCIENCE RESEARCH

B. Biomechanics

1. Should we investigate the biomechanics of:

	1 Extremely unimportant	2	3	4	5	6	7	8	9 Extremely important
a. Spinal manipulative therapy?	(1) ❑	(2) ❑	(3) ❑	(4) ❑	(5) ❑	(6) ❑	(7) ❑	(8) ❑	(9) ❑
b. Different manipulative and manual techniques, as a basis for comparison?	(1) ❑	(2) ❑	(3) ❑	(4) ❑	(5) ❑	(6) ❑	(7) ❑	(8) ❑	(9) ❑
c. Normal and abnormal joint biomechanics?	(1) ❑	(2) ❑	(3) ❑	(4) ❑	(5) ❑	(6) ❑	(7) ❑	(8) ❑	(9) ❑

If there is something missing from the list regarding biomechanics, please feel free to add your comments.

____

____

____

____

____

____

II. BASIC SCIENCE RESEARCH

C. Diagnostic

1. Should we investigate the following methods and techniques for the diagnosis of musculoskeletal pain:

	1 Extremely unimportant	2	3	4	5	6	7	8	9 Extremely important
a. Imaging e.g. MRI, PET, CT, ultrasound, flouroscopy, functional imaging?	(1) ❑	(2) ❑	(3) ❑	(4) ❑	(5) ❑	(6) ❑	(7) ❑	(8) ❑	(9) ❑
b. Postural and movement patterns?	(1) ❑	(2) ❑	(3) ❑	(4) ❑	(5) ❑	(6) ❑	(7) ❑	(8) ❑	(9) ❑
c. Thermal imaging and electromyography (EMG)?	(1) ❑	(2) ❑	(3) ❑	(4) ❑	(5) ❑	(6) ❑	(7) ❑	(8) ❑	(9) ❑
d. Eye movement patterns and visual perception?	(1) ❑	(2) ❑	(3) ❑	(4) ❑	(5) ❑	(6) ❑	(7) ❑	(8) ❑	(9) ❑

If there is something missing from the list regarding diagnostic, please feel free to add your comments.

____

____

____

____

II. BASIC SCIENCE RESEARCH

D. Theoretical concepts

1. Should we investigate the theories and/or theoretical models of:

	1 Extremely unimportant	2	3	4	5	6	7	8	9 Extremely important
a. The phases of spinal degeneration?	(1) ❑	(2) ❑	(3) ❑	(4) ❑	(5) ❑	(6) ❑	(7) ❑	(8) ❑	(9) ❑
b. The fixation/subluxation?	(1) ❑	(2) ❑	(3) ❑	(4) ❑	(5) ❑	(6) ❑	(7) ❑	(8) ❑	(9) ❑

If there is something missing from the list regarding theoretical concepts, please feel free to add your comments.

III. EDUCATION

1. Should we examine:

	1 Extremely unimportant	2	3	4	5	6	7	8	9 Extremely important
a. How student selection is conducted and how to select the best students?	(1) ❑	(2) ❑	(3) ❑	(4) ❑	(5) ❑	(6) ❑	(7) ❑	(8) ❑	(9) ❑
b. Curriculum design and implementation of curricula?	(1) ❑	(2) ❑	(3) ❑	(4) ❑	(5) ❑	(6) ❑	(7) ❑	(8) ❑	(9) ❑
c. Modes of delivery of postgraduate education?	(1) ❑	(2) ❑	(3) ❑	(4) ❑	(5) ❑	(6) ❑	(7) ❑	(8) ❑	(9) ❑
d. Philosophy of chiropractic care?	(1) ❑	(2) ❑	(3) ❑	(4) ❑	(5) ❑	(6) ❑	(7) ❑	(8) ❑	(9) ❑
e. Inter-professional learning?	(1) ❑	(2) ❑	(3) ❑	(4) ❑	(5) ❑	(6) ❑	(7) ❑	(8) ❑	(9) ❑

If there is something missing from the list regarding education, please feel free to add your comments.

____

____

____

____

____

____

IV. OTHER

1. Should we:

	1 Extremely unimportant	2	3	4	5	6	7	8	9 Extremely important
a. Promote chiropractic Ph.D's in academic institutions throughout Europe?	(1) ❑	(2) ❑	(3) ❑	(4) ❑	(5) ❑	(6) ❑	(7) ❑	(8) ❑	(9) ❑
b. Establish clinical research networks throughout Europe, such as Denmark and the	(1) ❑	(2) ❑	(3) ❑	(4) ❑	(5) ❑	(6) ❑	(7) ❑	(8) ❑	(9) ❑

	1 Extremely unimportant	2	3	4	5	6	7	8	9 Extremely important
UK?									
c. Initiate and promote collaborative research activities?	(1) ❑	(2) ❑	(3) ❑	(4) ❑	(5) ❑	(6) ❑	(7) ❑	(8) ❑	(9) ❑

We are also interested in your ideas as to how we can actually implement research into these topics. All comments are welcome.

____

____

____

____

____

____

Thank you very much for your participation. We will keep you informed on the progress of this study.

If you have answered all the questions and do not want to change answers, please click

the X below to complete and send the questionnaire.

Appendix 4. Comments at Phase 2, Round 1

**If there is something missing from the lists regarding effects of treatment, please feel free to add your comments.**	
No.	Comment
1	1. We should not be labeling treatments 'chiropractic' or otherwise. Treatments cross professional boundaries.<br>2. We need to be able to measure treatment effects objectively, not just from PROMS
2	non specific effects of treatment such as feeling reassured, being able to better cope with chronic symptoms or improved quality of life.
3	In addition to credibility in the reduction of pain and disability, we are often criticized for not being able to measure what we treat.... e.g. what is the manipulable lesion? measurement of function - movement patterns; EMG; etc. and the changes after SMT.
4	No. 3 mixes treatment effects on different age groups with two specific populations. Treatment effects on specific populations - e.g. diagnostic populations - are just as important to widen our 'scope of practice'.
5	chiropractic as holistic care, not just a crack of a joint. evaluation of the whole patient, taking into consideration the 3 keystones of health... the psychological aspect of the patient(e.g. the catastrophic mind set), the chemical aspect (e.g. the allergic, inflamed patient) and the mechanical aspect (e.g. the effect of prior injuries).
6	In 3. Again see previous answer in pilot here:<br>For 3 a and b adverse events, complications need to be studied as long as profession continues to make this a target population instead of an incidental population.
7	Predictors

**If there is something missing from the lists regarding prognostic research, please feel free to add your comments.**	
No.	Comment
1	Using variability of symptoms over time as an outcome rather than the traditional dichotomized MCIC at specific assessment points as dependant variables with possibly considering looking at early changes in independent variables (pain, locus of control, ROM etc) may prove more fruitful than more work along traditional lines.
2	Function..! does someone with persistent LBP have movement pattern disorders / EMG activity and does that change with SMT?
3	Ad 1 I do not believe it is up to the chiropractic profession to conduct research on the prognosis of other types of treatment.
4	personality type:<br>extremely motivated person: can't stop training despite injury and keeps hurting themselves.<br>Patient with no connection to their body, don't understand how to activate specific muscles.<br>Patient unwilling to change habits or train to get better.
5	do you mean b: clinical findings as distinct from validated outcome measures? If so then answer to b is not important. If you mean including pain and disability, then very important

**If there is something missing from the lists regarding prevalence/incidence/prevention/population studies of musculoskeletal conditions, please feel free to add your comments.**	
No.	Comment
1	I am not sure that work on the age group 20 - 65 has been exhausted and this contains the majority of those presenting to chiropractic clinics
2	preadolescents are a critical group and are no longer children. this is where intervention can make a difference, adolescents are very much more adults already. studies should look at how to prevent a pre-adolescent/adolescent with NP/LBP from becoming adult with more serious neck pain and LBP
3	see comments to pilot.

**If there is something missing from the list regarding issues of chiropractic practice, please feel free to add your comments.**	
No.	Comment
1	Need a comprehensive survey of practice that is representative and obtains information from patient files and not just from chiropractors' opinions of what is in them.
2	Exploring why chiropractors are at times engaging in prolonged courses of care. It may be found that reasons beyond the obvious are significant.
3	The influence of the quality of delivered treatment
4	ETHICS of practice

If there is something missing from the lists regarding anatomy and physiology, please feel free to add your comments.	
No.	Comment
1	Biomechanics
2	I believe we should look at these things, and it is my interest area ....however, I recognize the greater importance of sub-classification and epidemiology studies.

If there is something missing from the list regarding biomechanics, please feel free to add your comments.	
No.	Comment
1	as before.
2	I would say using a SMT on an adult can lead to a certain neurochemical cascade that can be measured and used for comparison, but an SMT used on a geriatric patient or a hot disc in an adult both of whom should have had gentler manual techniques would cause a different neuro-chemical cascade. As a physician I use my judgment all day long to decide which techniques are appropriate. we are back to subgroups and which types of techniques are appropriate. Research does not always allow for this type of subgrouping and therefore results may not be as positive as we would hope
3	see answer to 1C above

If there is something missing from the list regarding diagnostic, please feel free to add your comments.	
No.	Comment
1	Again, this is what I want to do, but the priority is lower....
2	The answers above are given based on the need to investigate the clinical use of the diagnostic modalities listed and not necessarily their value in research.
3	comment on 1A. These are studied amply by other groups. We need to prioritize funding allocation Comment on rest: if practiced commonly by chiro's, should be studied for relevance and validity

If there is something missing from the list regarding education, please feel free to add your comments.	
No.	Comment
1	student assessment especially in clinical settings
2	Effective administration practices; interprofessional education; creating curricula based upon the best EDUCATION evidence.
3	selection "d" is not clear: "philosophy of chiropractic care" is too vague
4	Not that it is unimportant - I just think it should be on the individual institutions agendas - not the research agenda for the profession.
5	subcomponents of being a "good" chiropractor: e.g., empathy psychomotor skills etc.
6	1 a. = impossible as long as there is a shortage of potential students.

We are also interested in your ideas as to how we can actually implement research into these topics. All comments are welcome.	
No.	Comment
1	I will send you a publication.. Many thanks
2	Set up centers of excellence for specific research themes at universities/institutions throughout Europe to maximize the attraction and use of funding as well as the provision of the necessary infrastructure and equipment. It does not mean that only people at that institution can undertake that research theme as through collaborative links it should facilitate others with interest in a similar area (but working at a different research) to contribute to that specific area of research.
3	You need to identify funding opportunities to implement the findings of this survey. Good luck and thanks.
4	Function is important to me - but being on the playing field, effectiveness and cost-effectiveness is more important for healthcare providers.<br>well done!
5	ECU could arrange focused and theme based workshops that aim to initiate collaborations within the chiropractic field, e.g. sports injuries, traumatology, paediatrics etc.
6	More countries should establish clinical research networks like KIP in Norway and Denmark, so clinical trials could be performed more easily, and so that researchers could work more on research and analysis than practical follow up, and data collection
7	See comments sent in pilot and to SM Rubinstein.

Comments at Phase 2, Round 2

Please provide any comments here (Regarding the items already agreed upon)	
No.	Comment
1	Important as it defines who we are.
2	Which items have been removed?
3	I agree with cost effectiveness and effects of chiropractic treatment on MSK pain. Prevention would also be a good area. After the Odense Conference I think the others are dead ends.
4	In order for chiropractors to remain in a leading academic and clinical position in the field of musculoskeletal medicine, they have a unique opportunity to provide new insights, ground/basic research concerning our (read everybody)understanding of the anatomical and physiological mechanisms of in particular chiropractic treatment of musculoskeletal conditions.<br>Hence, it is vital that our profession keeps in front in this area of research which to a certain extent provides basis for the very cornerstone of the chiropractic profession.<br>I would recommend that this is re-appraised as a vital area of research, although many practitioners may not find this having a great direct impact on their daily practice.
5	it is unclear which items have been removed

If there is something missing from the lists regarding effects of treatment, please feel free to add your comments.	
No.	Comment
1	Just a comment; the way I have "anchored" the weighting of my responses, is from a public health perspective. I may have found it easier to answer these questions if I could have arranged them by internal order of importance.
2	I think chiropractors should become more involved in "public health" initiatives. Back pain is a chronic, recurrent disease that affects all ages. Given its health burden, a population-based approach of health promotion targeting the same risk factors that are responsible for other chronic diseases would be useful (e.g., health promotion, physical fitness, etc).
3	pain and disability
4	1 c. above is essential for pediatric chiropractic care because utterly lacking; in adults less important because already quite thoroughly investigated.<br>3. a and b: see also 1c. The research is only necessary as long as the profession continues to place such an emphasis on treating this population, otherwise would not be.<br>3d is a non-starter: what is research on 'adults'? Almost every research project relevant to chiropractic to date has been on adults.......

If there is something missing from the lists regarding prognostic research, please feel free to add your comments.	
No.	Comment
1	The identification of mechanical subgroups seems most important for chiropractors
2	Comment: to study for example imaging appears with the knowledge we have today to be irrelevant in relation to chiropractic treatment. However in some circles imaging is used and abused, which means that it would be important to do some basic research in this area, mainly to stop abusive and illogical, implausible behavior. Difficult to put it in its "real" place, in other words.
3	What about the social determinants of health and recovery (e.g., cultural, legislation on insurance systems, early life risk factors).
4	fMRI is one of the few bright spots for future research
5	For 2 d I include diagnostic ultrasound. Else it would get a lower rating from me.
6	association between inflammatory conditions and prognosis

If there is something missing from the lists regarding prevalence/incidence/prevention/population studies of musculoskeletal conditions, please feel free to add your comments.	
No.	Comment
1	As for the incidence and prevalence of this that and the other, there are probably no big surprises lurking in the forest of data. Starts early remains at about the same level. Perhaps it could be possible to investigate if it is the same things that hurt throughout life or if there are different things.
2	prevention being the most useful use of research capacity.
3	prevention<br>ergonomic interventions in school for teenagers, implementing availability of standing in classrooms, lockers and rolling bags
4	As stated in first round: where research is or can be carried out by other groups with oooooodles more $$$$ than chiropractors have, let it be!

	Only in areas where chiropractic can add a variable not examined elsewhere should we consider funding a project.

**If there is something missing from the list regarding issues of chiropractic practice, please feel free to add your comments.**	
No.	Comment
1	Incidence and severity of unexpected and unpleasant consequences of care - in this context possibly looking not at the very severe but at the mild and moderate.<br>This may be felt important because most studies of outcomes show very similar results comparing different interventions for MSK conditions. However if some are achieving this will fewer unpleasant side effects (ache, stiffness, tiredness etc) then they might be considered preferable (by the patient).
2	Ethics should be examined in an expert ethics committee and not in research

**If there is something missing from the lists regarding anatomy and physiology, please feel free to add your comments.**	
No.	Comment
1	I think the investigation of anatomical and/or (neuro)physiological basis for MS pain and disability and function is already being investigated outside the chiropractic profession and with limited resources we could spend time and money on other areas (see previous questions).
2	No 2: This question is very difficult to answer as I am not sure what you mean by 'chiropractic treatment'. Would suggest you specify what you mean.<br>Secondly, it is BAD questionnaire technique that I cannot continue without answering all questions. Now I have put something down that I do not agree with!!!!!!
3	it is not that the anatomy and neuro are irrelevant, just that the basic science and medical science should be performed by those with the spare capacity to do it.

**If there is something missing from the list regarding biomechanics, please feel free to add your comments.**	
No.	Comment
1	Perhaps first see if there is an effect of SMT...
2	it's not biomechanical - it's neurological..!
3	Regarding 1c. Particularly in regard to if abnormal joint biomechanics have any correlation with symptoms, function of daily living, work capability, QOL etc.

**If there is something missing from the list regarding diagnostic, please feel free to add your comments.**	
No.	Comment
1	Yes; I think that knowledge of mechanisms is largely unknown or at least that we do not understand that literature very well.
2	Whatever is promising from<br>the literature
3	fluoroscopy misspelled above..... :-)

**If there is something missing from the list regarding theoretical concepts, please feel free to add your comments.**	
No.	Comment
1	Yeah; the fixation/subluxation but not from this simplistic perspective. The "effect" of the SMT needs addressing to separate placebo from true effect and to separate the effect of SMT regardless an underlying "lesion" and when/if there is indeed an underlying "lesion".

**If there is something missing from the list regarding education, please feel free to add your comments.**	
No.	Comment
1	Effectiveness of curricula
2	Effectiveness of different types of educational styles in relation to different types of learning needs
3	I don't know what you mean by "philosophy of chiropractic care." If it is the old school innate intelligence stuff my answer is extremely unimportant. If it is a conceptual model for the chiropractic profession, then it is extremely important.

4	I think these are important issues, but not research.
5	1. a. would be important in ideal world, but as long as chiropractic schools are struggling to fill seats, any results from such studies will not be applied

**We are also interested in your ideas as to how we can actually implement research into these topics. All comments are welcome.**	
No.	Comment
	Those that collaborate always win.
	why have tutors in the academic population who are only there based on experience? students develop the perception that research is unnecessary and irrelevant if the tutors do not perform research.

Appendix 5. Phase 2, Round 3

Consensus on the importance of a number of items has now been achieved.

However we should like you to state which of these 19 items you find most important by rating 5 of them as the most important, second most important etc.

Below you find a list with the 19 items.

Please read the entire list carefully and note the numbers of the ones you consider most important in the table at the bottom of the sheet.

Epidemological and clinical research. Treatment effects in the area:

1. Dose response and frequency of treatment.

2. Cost-effectiveness.

3. Long-term effects of treatment.

4. Identification of subgroups likely to respond to treatment.

5. Effects and responses upon quality of life.

6. Effetcs and responses upon function and performance.

7. Effects and responses upon treatment on musculoskeletal pain.

Epidemological and clinical research. Treatment and effects in specific populations:

8. Adolescents.

9. Geriatrics.

10. Working population, work disability prevention and injured workers.

Epidemological and clinical research. Prognostic research:

11. In the clinical course of musculoskeletal pain in chiropractic population.

12. In clinical findings as predictors of outcome.

13. In predictors of outcome; the interaction between biological and psychosocial variables.

Epidemological and clinical research. Prevalence, incidence and prevention:

14. Prevention of musculoskeletal pain in primary and secondary care.

15. Musculoskeletal conditions in a working population.

Basic science research:

16. The anatomical and/or neurophysiological basis of chiropractic treatment on musculoskeletal pain.

Other:

17. Promotion of chiropractic Ph.D.'s in academic institutions throughout Europe.

18. Establishing clinical research networks throughout Europe.

19. Initiation and promotion of collaborative research activities.

The item I consider to be the most important. Item no.	__
The item I consider to be second most important. Item no.	__

The item I consider to be third most important. Item no.	__
The item I consider to be fourth most important. Item no.	__
The item I consider to be fifth most important. Item no.	__

Thank you very much for your participation. We will keep you informed on the progress of this study.

If you have answered all the questions and do not want to change answers, please click the X below to complete and send the questionnaire.
